# Epigenetics of Fear, Anxiety and Stress – Focus on Histone Modifications

**DOI:** 10.2174/1570159X21666230322154158

**Published:** 2023-09-01

**Authors:** Marco A. Ell, Miriam A. Schiele, Nicola Iovino, Katharina Domschke

**Affiliations:** 1 Department of Psychiatry and Psychotherapy, Medical Center, University of Freiburg, Faculty of Medicine, University of Freiburg, Germany;; 2 Department of Chromation Regulation, Max Planck Institute of Immunobiology and Epigenetics, Freiburg, Germany;; 3 Center for Basics in NeuroModulation, Faculty of Medicine, University of Freiburg, Freiburg, Germany

**Keywords:** Epigenetics, chromatin, histone, HDAC, anxiety, fear, stress, Bdnf

## Abstract

Fear-, anxiety- and stress-related disorders are among the most frequent mental disorders. Given substantial rates of insufficient treatment response and often a chronic course, a better understanding of the pathomechanisms of fear-, anxiety- and stress-related disorders is urgently warranted. Epigenetic mechanisms such as histone modifications - positioned at the interface between the biological and the environmental level in the complex pathogenesis of mental disorders - might be highly informative in this context. The current state of knowledge on histone modifications, chromatin-related pharmacology and animal models modified for genes involved in the histone-related epigenetic machinery will be reviewed with respect to fear-, anxiety- and stress-related states. Relevant studies, published until 30^th^ June 2022, were identified using a multi-step systematic literature search of the PubMed and Web of Science databases. Animal studies point towards histone modifications (*e.g*., H3K4me3, H3K9me1/2/3, H3K27me2/3, H3K9ac, H3K14ac and H4K5ac) to be dynamically and mostly brain region-, task- and time-dependently altered on a genome-wide level or gene-specifically (*e.g*., *Bdnf*) in models of fear conditioning, retrieval and extinction, acute and (sub-)chronic stress. Singular and underpowered studies on histone modifications in human fear-, anxiety- or stress-related phenotypes are currently restricted to the phenotype of PTSD. Provided consistent validation in human phenotypes, epigenetic biomarkers might ultimately inform indicated preventive interventions as well as personalized treatment approaches, and could inspire future innovative pharmacological treatment options targeting the epigenetic machinery improving treatment response in fear-, anxiety- and stress-related disorders.

## 1 INTRODUCTION

Fear-, anxiety- and stress-related disorders such as specific phobias, agoraphobia, social anxiety disorder, generalized anxiety disorder, panic disorder or post-traumatic stress disorder (PTSD) constitute the most frequent mental disorders with a cumulative 12-month prevalence of ~16% and confer a substantial individual as well as socioeconomic burden [1-6]. The first-line treatment of fear-, anxiety- and stress-related disorders comprises psychopharmacological options as well as psychotherapy. However, non-response rates are considerable, and in about 30% of the cases, the disorders take an intermittently relapsing or even chronic course [7].Thus, a better understanding of the pathomechanisms of fear-, anxiety- and stress-related symptoms is urgently warranted and can be expected to inform innovative and personally tailored treatment approaches.

The etiology of fear-, anxiety- and stress-related disorders is complex with an interaction of biological factors and environmental influences [3, 5]. Epigenetic mechanisms comprising histone modifications, deoxyribonucleic acid (DNA) methylation as well as non-coding RNAs (ncRNAs) have been suggested to be positioned at the interface between the biological and the environmental level and to dynamically broker adaptation or maladaptation, respectively, to environmental conditions or challenges [8-10]. Thus, epigenetic mechanisms constitute prime candidates in the search for fear-, anxiety- and stress-related disorder biomarkers informing indicated preventive interventions as well as personalized treatment approaches, and possibly also inspiring future innovative pharmacological treatment options targeting the epigenetic machinery.

As an introduction, the present paper provides a propaedeutic overview of the terminology and basic mechanisms of epigenetics spotlighting histone modifications. The subsequent main focus will be on reviewing the current state of knowledge on histone modifications, chromatin-related pharmacology and animal models mutant for genes involved in the histone-related epigenetic machinery with respect to fear-, anxiety- and stress-related states in an effort to complement and extend existing reviews [11-13]. For a comprehensive overview of studies on the role of DNA methylation and ncRNAs in this context several previous reviews may be referred *e.g*., [8, 9, 11, 14-23].

### 1.1 Propaedeutics on Epigenetics

**Fig. (1):**
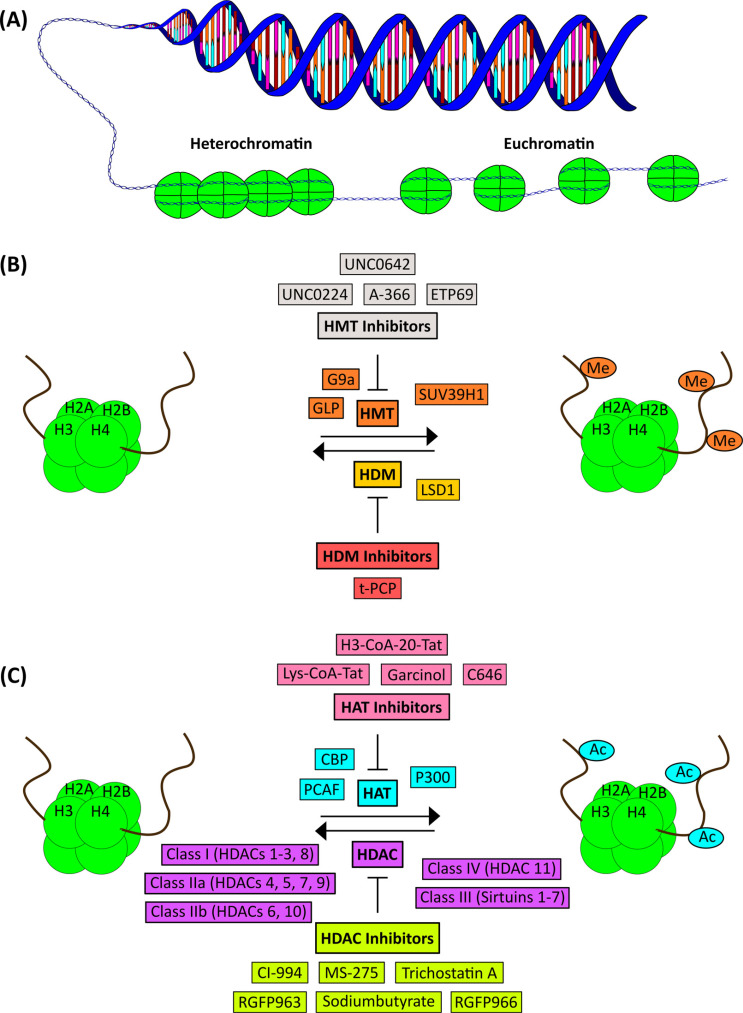
Chromatin modifications and regulators targetable by currently available chemical compounds. Schematic illustration of (**A**) the DNA double-strand wrapped around histone (H2A, H2B, H3, H4) octamers composed of two H2A-H2B dimers and two H3-H4 dimers to form the primary structure of chromatin, which can exist in a compact, *i.e*. closed state (‘heterochromatin’), or in an open state (‘euchromatin’); (**B**) mechanisms of histone methylation (HMT: histone methyltransferase) and demethylation (HDM: histone demethylase) as well as exemplary pharmacological HMT and HDM inhibitors; (**C**) mechanisms of histone acetylation (HAT: histone acetyltransferase) and deacetylation (HDAC: histone deacetylase) as well as exemplary pharmacological HAT and HDAC inhibitors; for further details and abbreviations see main manuscript (1.1. and 3.3.).

The genetic structure rests on DNA which is composed of nucleotides consisting of deoxyribose, a phosphate group and the four nitrogenous bases (nucleobases) guanine (G), cytosine (C), adenine (A) and thymine (T) that form specific pairs of A/T and G/C on opposite strands of the DNA double helix. Sequences of these nucleic acids directly encode genetic information. The DNA strands are wrapped around histone (H2A, H2B, H3, H4) octamers composed of two H2A-H2B dimers and a H3-H4 tetramer forming a packaging unit called nucleosome. Nucleosomes build threads or fibers with so-called linker DNA organized by the linker histone H1 in between them to form the primary structure of chromatin. Depending on the packaging density, chromatin can exist in a compact, *i.e*. closed state (‘heterochromatin’), which limits the accessibility of the genomic DNA for proteins like transcription factors (TFs) and thus lead to a transcriptionally ‘silent’ state. The looser, *i.e*. open state (‘euchromatin’) facilitates binding of the transcriptional machinery and subsequent gene transcription (Fig. **1A**) [24]. Chromatin is organized in a hierarchical fashion inside the nucleus, and the highest hierarchical level results in the chromosomes [25].

The term epigenetics literally translated means ‘above genetics’. This term was first introduced in 1942 by the embryologist Conrad Waddington who defined it as “the branch of biology that studies the causal interactions between genes and their products which bring the phenotype into being” [26]. Nowadays, the study of epigenetics is concerned with heritable changes that influence gene expression without affecting the DNA sequence itself. Epigenetic factors act on different cellular levels and can have either activating or repressing properties on gene expression profiles [27]. Such alterations can be transferred through mitosis to the next cell generation or through meiosis between organismal generations [28, 29]. Epigenetic change is a regular and natural occurrence mostly observed during early embryonic development and cell differentiation but remains partly malleable throughout the lifespan. Epigenetic processes have been suggested to be related to a vast amount of biological processes as well as environmental stressors, chemicals, drugs, aging, diet, mental state, social interaction or physical workouts [29, 30]. The three major players initiating and sustaining epigenetic change are histone modifications, DNA methylation as well as ncRNAs along with histone variants such as H3.3 and H2A.Z (for review see [31].

Post-translational histone modifications crucially govern DNA packaging density around histone proteins and mainly comprise methylation or acetylation (Figs. **1B** and **1C**), but also for instance phosphorylation, GlcNAcylation, biotinylation, butyrilation, citrullination, crotonylation, carbonylation, ubiquitination, SUMOylation, ADP-ribosylation or isomerization targeting terminal tails of histone proteins [32]. The nomenclature of histone modifications starts with a capital ‘H’ for ‘histone’ followed by a number indicating the amino acid position of the modification, and ends with the type (*e.g*., acetylation) of the modification itself. For example, acetylation at lysine 27 of histone 3 would read ‘H3K27ac’. Histone modifications are dynamically deposited or removed at the histone tails by enzymatic ‘writers’ or ‘erasers’, after being recognized by ‘reader’ proteins [33]. Histone modification ‘writers’ comprise histone methyltransferases (HMTs) or histone acetyltransferases (HATs) that confer methylation or acetylation to tails of histones, respectively (Figs. **1B** and **1C**). HMTs exemplarily comprise the euchromatin histone-lysine N-methyltransferase 2 (G9a), the G9a/G9a-like protein (GLP) and the Suppressor of Variegation 3-9 Homolog 1 (SUV39H1). Prominent representatives of HATs are the E1A binding protein P300, the CREB binding protein (CBP) and the p300/CREB-binding protein-associated factor (PCAF). The counterparts of ‘writers’ are termed ‘erasers’ and remove methyl or acetyl groups from histone tails. Erasers comprise histone demethylases (HDMs) such as the lysine-specific histone demethylase 1A (LSD1) and various classes of histone deacetylases (HDACs; classes I [HDACs 1-3 and 8], IIa [HDACs 4, 5, 7 and 9], IIb [HDACs 6 and 10], III [Sirtuins 1-7], IV [HDAC 11]) (Figs. **1B** and **1C**). Methylation mainly occurs at lysines of histone H3. Lysine methylation can have either activating or repressing effects on gene transcription depending on the methylation site [34]. Additionally, lysines can be mono-, di- or tri-methylated conferring either activating or repressing transcriptional effects. For example, H3K4me3 is involved in active gene transcription, while H3K9me3 or H3K27me3 constitute repressive marks [35, 36]. Arginine residues on H3 and H4 can also be methylated, and this modification is generally linked to transcriptional activation. Acetylation of histone tails occurs at lysine residues, which thereby lose their positive charge resulting in the neutralization of the charge on the respective histone and consequently in the weakening of the binding to the negatively charged DNA. This weaker histone-DNA interaction contributes to an ‘open’ euchromatin state significantly increasing gene expression [37]. For a summary of the most prominent histone modifications and their studied function as well as preferred locations (Table **1**).

## 2 METHODS

A multi-step systematic literature search of the PubMed and Web of Science databases was performed by independent researchers ME and KD applying the following broad search terms: (“histone” OR “histone modification” OR “acetylation”) AND (“anxiety” OR “fear” OR “extinction” OR “startle” OR “stress” OR “anxiety disorder*” OR “stress-related disorder” OR “stress-related” OR “post-traumatic stress disorder” OR “PTSD” OR “panic disorder” OR “panic” OR “social phobia” OR “social anxiety disorder” OR “agoraphobia” OR “specific phobia*”). Identified articles published until 30^th^ June 2022 were screened based on title and abstract reading. Only peer-reviewed studies published in English reporting on original research or review articles were considered eligible to be included in the present review. After the exclusion of articles irrelevant to the topic investigated, full texts of the remaining articles were further assessed for inclusion. Searching reference lists of selected articles and pertinent review articles revealed additional eligible articles. Disagreements in search and selection were resolved through discussion and consensus.

## 3 RESULTS

### 3.1 Histone Modifications in Animal Models of Fear and Anxiety

Paradigms such as contextual fear conditioning, partly combined with single-prolonged stress, auditory fear conditioning or fear extinction following conditioning in animals serve as mechanistic models for mostly fear- and anxiety-related disorders and their treatment [38], but have also been shown to constitute valid models for stress-related disorders such as PTSD in humans [39]. Epigenetic studies analyzing dynamic chromatin remodeling processes based on histone modifications in animal models have mostly been conducted on tissue harvested from brain regions centrally engaged in the brain fear circuit including the hippocampus, entorhinal cortex, anterior cingulate cortex (ACC), prefrontal cortex (PFC), amygdala or bed nucleus of the stria terminalis (BNST) [40, 41].

Eight studies investigated the role of histone modifications related to contextual fear conditioning. Following a standard contextual fear conditioning paradigm, H3K4me3 was observed to be significantly increased in the hippocampus, entorhinal cortex and ACC one hour after conditioning [42-45]. There are, however, conflicting results on whether increased H3K4me3 is persistent over a time period of 24 hours [44], returns to baseline [43], or even decreases below naïve levels after 24 hours [45]. Interestingly, in the CA1 hippocampal area as well as the ACC, H3K4me3 – accompanied by increased H3K9ac and decreased H3K27me3 – was only increased in genic regions, while in intergenic regions H3K4me3 was decreased along with increased H3K27me3 [42]. Contextual fear conditioning has furthermore been shown to lead to temporary increases of global H3K14ac levels [46] and transiently increased global levels of H4K5ac predominantly at promoter regions [47] one hour after conditioning. At random time points within an eight-minute training period, contextual fear conditioning was accompanied by globally increased H2BK5ac, H3K9K14ac and H4K12ac levels in the hippocampus, with H2BK5ac and H3K12ac being most specifically linked to the context-shock association, *i.e*. to learned fear [48]. On a candidate gene level, most studies have focused on the gene coding for the brain-derived neurotrophic factor (BDNF), which is crucially involved in brain development, neural plasticity, memory formation and particularly the acquisition, consolidation and extinction of conditioned fear [49-51]. Two hours after contextual fear conditioning, partly in interaction with single prolonged stress, levels of H3ac and H4ac at *Bdnf* promoters I and IV were found to be increased in the hippocampus along with elevated total Bdnf mRNA levels [52]. Accordingly, another study observed *Bdnf* promoter IV, histone H3 acetylation as well as phosphoacetylation to be increased two hours after contextual fear conditioning along with increased Bdnf exon IV mRNA and decreased DNA methylation at *Bdnf* exons I and IV. At *Bdnf* promoter II, however, H4 acetylation was discerned to be decreased [53].

Retrieval of fear memory after contextual fear conditioning evoked differential epigenetic changes depending on the time frame of memory formation and the respective brain area. After retrieval of a recent (one hour) contextual fear memory, H3K4me3 was found to be increased in the hippocampal CA1 region both on a global level and in the coding region of the *Npas4* but not the *C-fos* gene. Yet, following the retrieval of a remote fear memory 30 days after memory acquisition, increased H3K4me3 levels were observed in CpG-enriched coding regions of the *C-fos* gene in the ACC [44] suggesting differential and brain region-specific gene targets of increased H3K4 trimethylation following retrieval of recent or remote fear memory, respectively.

Two studies report on histone modifications in the context of auditory fear conditioning paradigms. On a genome-wide level, H3K9me2 was increased in the lateral amygdala both one hour and 24 hours after conditioning [54]. On a candidate gene level, auditory fear conditioning was furthermore shown to lead to increased H3ac levels at *Bdnf* promoters I and IV in the PFC, while fear extinction training reversed increased H3ac at *Bdnf* promoter I to below naïve levels and was furthermore associated with increased H4ac at *Bdnf* promoter IV along with increased Bdnf exon I and IV mRNA expression in the PFC [55]. In a similar vein, on a genome-wide level, following fear learning H3ac and H4ac increased in the amygdala and the prelimbic PFC, but not in the infralimbic PFC, while extinction learning led to increased H3ac in both the prelimbic and infralimbic PFC and increased H4ac in the infralimbic PFC only [56]. In addition, another study on histone modifications in BNST sub-regions observed fear conditioning to be accompanied by increased H3ac and H4ac in the anteromedial and ventrolateral BNST, while following extinction H4ac increased in the anterolateral BNST specifically [57].

Finally, one study focused on the quality of fear extinction, measured two hours after extinction, dependent on the time of extinction after memory acquisition. Immediate extinction ten minutes after fear memory acquisition did not lead to retention of fear extinction whereas delayed extinction 24 hours after fear memory acquisition led to the retention of fear extinction, measured by the relative freezing response to the previously conditioned fear. In both extinction paradigms, global levels of H3K9ac were increased in the prelimbic and infralimbic PFC along with elevated levels of c-Fos and CBP in both regions. However, delayed extinction resulted in a stronger increase in H3K9ac and CBP levels in the infralimbic PFC as compared to immediate extinction (Table **2**) [58].

### 3.2 Histone Modifications in Animal Models of Acute and (Sub-)Chronic Stress

The role of epigenetic mechanisms in moderating the response to acute or (sub-)chronic stressful environmental influences and thus ultimately in possibly conferring risk of fear-, anxiety- and stress-related disorders has been investigated in animal models during different time windows of development (pre-conception, pre-/post-natal, juvenile, adult age) in fear- or anxiety-relevant brain regions (*e.g*., hippocampus, limbic system, cortex) using paradigms such as restraint stress, social defeat, forced swimming, predator stress, acute glucocorticoid treatment, chronic unpredictable stress or social isolation [59].

#### 3.2.1 Acute Stress

Six studies were identified that explicitly investigated the effect of acute stress on histone modifications in rodent models (Table **3**). Following acute restraint stress for 30 minutes, increased H3K9me3 as well as decreased H3K27me3 and H3K9me1 were observed on a genome-wide level in hippocampal tissue [60]. Against the background of a history of chronic restraint stress, two and 24 hours after acute restraint the histone acetyltransferase P300 was found to be involved in dynamic up-regulation (two hours) and down-regulation (24 hours), respectively, of metabotropic glutamate receptor 2 expressions in the hippocampus *via* differential acetylation of H3K27 bound to the metabotropic glutamate receptor 2 gene (*Grm2*) promoter [61]. Another study investigating the effects of a two-hours-restraint stress paradigm on histone modifications at the *Bdnf* gene discerned a significant decrease in acetylated H3 at *Bdnf* gene promoters I, IV and VI two hours post-stress along with a decrease in total Bdnf mRNA levels as well as Bdnf exon I-, and exon IV-specific mRNA, which, however, could both not be observed 24 hours post-stress [62]. Following the repeated social defeat, global levels of H3 acetylation in the hippocampus, amygdala, and PFC increased 30 minutes after the last defeat [63]. In a study applying forced swimming, predator, ether exposure as well as cold stress, only forced swimming and predator stress evoked increased genome-wide H3-phosphorylation [64]. Finally, acute glucocorticoid treatment was shown to decrease H3K9me3 on a genome-wide level at B2 short interspersed nuclear elements in the hippocampus [65].

#### 3.2.2 (Sub-)Chronic Stress

(Sub-)chronic stress can be modelled in animals by the application of restraint stress over longer time periods (Table **4**). After subchronic restraint stress for seven days, hippocampal genome-wide H3K4me3 and H3K27me3 levels were observed to be decreased and H3K9me3 to be increased [60]. Subchronic restraint stress was furthermore shown to decrease levels of H3K9me2 bound to the promoter region of the gene coding for the E3 ubiquitin-protein ligase, also known as neural precursor cell expressed developmentally down-regulated protein 4 (*Nedd4-1)* and to increase general HDAC2 levels in the PFC [66].

Conversely, genome-wide levels of hippocampal H3K4me3 increased and H3K9me3 decreased following the application of chronic restraint stress applied for 21 days [60]. Chronic restraint stress lasting for 29 days was furthermore accompanied by decreased genome-wide histone H3K9 and H4K5 acetylation levels in hippocampal neurons along with an upregulation of HDAC2 colocalized with glucocorticoid receptors [67].

Another model of chronic stress comprises social defeat over longer time periods (Table **4**). Immediately and one day after a ten-day social defeat paradigm, HDAC2 and HDAC5 levels were found to be decreased in the paraventricular nucleus and the nucleus accumbens, respectively [68, 69]. Additionally, ten days after a ten-day social defeat stress paradigm, decreased global H3K9me2 levels along with decreased levels of the associated writer enzymes G9a and Glp and the repressive chromatin modifier Suv39h1 were observed in the nucleus accumbens [70]. In a related experiment, 24 hours and ten days after ten-day social defeat stress, global H3K14ac significantly increased in the nucleus accumbens along with a significant decrease in HDAC2 mRNA levels [71]. Twenty-eight days after ten-day social defeat stress, H3K27me2 levels were discerned to be increased both on a candidate gene level at the *Bdnf* P3 and P4 promoter in the hippocampus accompanied by downregulation in total *Bdnf* and particularly *Bdnf* III and IV mRNA levels [72] as well as on a genome-wide level in the nucleus accumbens [73]. In line with these findings, a similar model of chronic social stress (social isolation) investigating genome-wide histone modifications also 28 days after the stressor revealed H3K27me2 and H3K9me2 levels in the nucleus accumbens to be increased [73]. Finally, immediately following extended social defeat over 21 days, genome-wide H3K9ac was observed to be decreased in the hippocampus, accompanied by increased histone acetyltransferases and accordingly decreased histone deacetylases in the cortex. Three weeks after the last social defeat, H4K12ac, H3K4me3 and histone acetyltransferase activity were increased in the hippocampus [74].

Three studies were identified that applied chronic variable or mild stress for 14 to 15 days. Here, a significant genome-wide decrease in both H4K12ac and phospho-acetylation of H3 (Lys9/Ser10) in the CA3 region and dentate gyrus of the hippocampus was revealed [75]. Accordingly, a study combining chronic variable stress with subsequent acute stress for 30 minutes observed that chronic variable stress reduced genome-wide H4K12ac levels in the hippocampus, while acute stress both on its own or following chronic variable stress went along with increased H4K12ac [76]. Furthermore, chronic variable mild stress resulted in a sex-specific upregulation of histone acetyltransferase CBP mRNA levels in the BNST in female animals and a decrease in histone deacetylase 5 (HDAC5) mRNA levels in the central nucleus of the amygdala in males [77].

Chronic unpredictable stress for 21 to 28 days was shown to decrease H3K9ac levels along with increased HDAC5 expression in the hippocampus on a genome-wide level [78], and to decrease H3K9me3 on a candidate gene level at the promoter of the corticotropin-releasing hormone receptor 1 (*Crh1*) gene in the hypothalamus [79]. In the specific context of pregnancy, chronic unpredictable stress applied four weeks prior to, and then throughout pregnancy resulted in a global downregulation in H3K14ac in the hippocampus of the offspring with a stronger effect in female offspring, along with a decrease in spatial memory [80]. Accordingly, gestational stress induced by a combination of restraint and 24-hours light disturbance to pregnant mice also induced decreased genome-wide H3K14ac as well as increased expression of DNMT1, HDAC1 and HDAC2 in the offspring [81]. Repeated cross-fostering in mice, an early interference with the maternal environment, was on a descriptive level linked to mostly increases in H3K4me3 and H3ac as well as predominantly decreased H3K27me3 levels in the medulla oblongata in the offspring, possibly driving an oversensitivity to carbon dioxide [82].

Finally, in an animal model using prolonged emotional and pain stress for 15 days an increased activity of Methyl-CpG Binding Protein 2 (MeCP2), which acts as a transcriptional repressor in complex with histone deacetylases, as well as an increase of H4ac were discerned at two weeks post-stress and were still observable two months post-stress [83].

### 3.3 Pharmacological Compounds Targeting the Histone Acetylation/Methylation Machinery

Epigenetic mechanisms involving chromatin have been shown to be implicated in the mode of action of several established psychopharmacological agents such as valproic acid (VPA), which has been shown to decrease anxiety symptoms *e.g*. [84-87], or serotonin re-uptake inhibitors (SSRIs)/ serotonin and norepinephrine re-uptake inhibitors (SNRIs), which are approved for the treatment of anxiety- and stress-related disorders [88, 89]. In rodent models, innovative pharmacological compounds mainly targeting erasers (HDACs, HDMs) but also writers (HMTs, HATs) (Figs. **1B** and **1C**) are currently under research regarding anxiety, fear- and place memory formation or consolidation as well as the extinction of fear-related memories [90-92]. A selective review of these approaches is provided below and in Table **5**.

#### 3.3.1 VPA/SSRIs and SNRIs

In an animal model, VPA, known to exert inhibitory effects on HDACs, has been shown to increase the efficiency of extinction training and long-term extinction memory along with elevated acetylation levels of H4 at *Bdnf* promoters I and IV [55]. Furthermore, treatment with VPA before stress-related memory formation in a two-day forced swimming test resulted in increased memory consolidation along with a significant decrease in ERK-phosphorylation and Bdnf protein levels in the ventrolateral orbital cortex on the following days [93]. In line with these findings, another study testing the effects of pre-test treatment with VPA on memory consolidation, reconsolidation and extinction of cued-fear after seven days of memory acquisition, VPA applied before each paradigm was observed to enhance consolidation, reconsolidation and extinction of long-term memory for conditioned fear, respectively [94]. In a mouse model of PTSD applying a 40-day regimen of predator exposure/ psychosocial stress, elevated superoxide and reactive oxygen species levels were observed in whole blood along with a global increase in HDACs in the PFC. These effects returned to naïve levels when VPA was administered daily for 30 days following the stress regimen [95]. Administration of VPA furthermore prevented increased HDAC5 expression as well as anxiety- and depression-like behavior induced by chronic unpredictable stress (see 3.3.2. [78]) along with a rescue of decreased H3K9 and H4K12 acetylation [78].

Treatment with the SSRI fluoxetine immediately before each individual restraint during 21-day restraint stress blocked the decrease of hippocampal genome-wide H3K9me3 24 hours post-stress, that occurred without fluoxetine treatment [60]. In a study consecutively applying 21 days of corticosterone treatment accompanied by 14 days of fluoxetine treatment starting by day eight of corticosterone treatment, an increase in anxiety- and depression-like behavior invoked by corticosterone stress was prevented by the application of fluoxetine. Further, all the biochemical changes invoked by chronic corticosterone treatment, like increased serum corticosterone and adrenocorticotropic hormone levels and alterations in several immunological markers were not observed in fluoxetine-treated animals [96]. Further, continuous treatment with the SNRI venlafaxine for 28 consecutive days was observed to prevent an increase in hippocampal HDAC5 expression and subsequent decreases in H3K9ac, H3K14ac and H4K12ac observed after 30 days of chronic unpredicted stress, leading to a significant improvement in anxiety- and depression-like behaviors [97]. In another rodent model of fear extinction following chronic unpredicted stress, treatment with venlafaxine was accompanied by decreased H3K9me2 along with anxiolytic effects [98].

#### 3.3.2 Experimental Compounds Targeting Erasers (HDAC, HDM)

Broad spectrum HDAC inhibitors such as sodium butyrate (NaBu) or trichostatin A have quite consistently been reported to enhance fear conditioning, memory formation and anxiety-like behavior. For instance, NaBu prompted a strong long-term potentiation at Schaffer-collateral synapses *in vitro* along with an increase in genome-wide acetylation of histones H3 and H4 in area CA1 of the hippocampus, and resulted in increased freezing behavior 24 hours, but not immediately after contextual fear conditioning when administered *in vivo* one hour prior to conditioning relative to saline treatment [46]. Likewise, systemic NaBu administration one hour prior to fear conditioning in nitric oxide synthase knockout mice led to genome-wide increases in acetylation of histones H3 and H4 in the hippocampus and amygdala as well as a significant increase in contextual fear conditioning 24 hours and seven days, respectively, but not immediately after training. However, no effect of NaBu treatment on fear conditioning and retention was observed in wildtype mice [99]. Trichostatin A was shown to enhance memory consolidation of inhibitory avoidance along with increased hippocampal Bdnf levels when infused into the rat basolateral amygdala 1.5, 3 or 6 hours post-training, but not when given pre-training [100], and to enhance long-term object-location memory, again when given post fear conditioning [101]. Furthermore, acute administration of NaBu resulted in increased anxiety-like behavior in the novelty-induced hypophagia paradigm accompanied by an increase in global acetylation levels of H4 in the hippocampus 30 minutes after NaBu treatment [102]. Chronic treatment with NaBu twice daily for 21 days, however, did not lead to any anxiogenic behavior in the novelty-induced hypophagia paradigm and was accompanied by a global decrease in hippocampal H4 acetylation levels [102]. Accordingly, chronic bilateral infusion of trichostatin A for seven days – starting at day 8 of 15-day corticosterone stress elicited by the implantation of corticosterone micropellets in the central nucleus of the amygdala - was able to attenuate a number of corticosterone-induced effects such as increased anxiety-like behavior, decreased mechanical somatic sensitivity, a genome-wide downregulation of H3K9ac, increased c-Fos, CRF and SIRT6 expression and decreased glucocorticoid receptor expression [103]. Regarding the role of broad-spectrum HDAC inhibitors in fear extinction, four independent studies reported mainly extinction-enhancing effects: Systemic NaBu administration was observed to increase genome-wide acetylation of histone H4 in the hippocampus and amygdala as well as H3 acetylation in the amygdala, and application prior to fear conditioning appeared to accelerate cued, but not contextual fear extinction - while not affecting fear acquisition (see above) - after five days of extinction training [99]. Accordingly, systemic administration of NaBu or intrahippocampal injection of trichostatin A prior to a short three-minute extinction training showed improved fear extinction usually only observed after a longer, 24-minute extinction session [104]. A similar result was obtained by intra-hippocampal NaBu injection following weak extinction - otherwise leading to an insufficient fear extinction - that induced improved levels of extinction along with increased infralimbic H3K14ac levels and enhanced c-Fos expression comparable to strong extinction effects [105]. Subcutaneous administration of NaBu for seven days was furthermore shown to alleviate impaired learning and memory in rats exposed to a single prolonged stress procedure and to enhance the extinction of newly formed fear memory [106].

Infusion of the HDAC1 and HDAC2 inhibitor suberoylanilide hydroxamic acid (SAHA), also known as vorinostat, into the basolateral amygdala over seven days resulted in a significant induction of global H3 and H4 acetylation along with increased anxiety-like behavior in the open-field test and impairments in cue-dependent memory in a fear-conditioning paradigm [107]. In another study testing vorinostat on fear consolidation and extinction when injected intraperitoneally after one day of fear conditioning or after the second day of extinction training following one day of fear conditioning, vorinostat was also shown to increase global acetylation levels of H3 and H4 after two hours while leading to facilitated fear extinction [108]. In a rat model of single prolonged stress followed by contextual fear conditioning, vorinostat injected after two days of fear extinction training led to enhanced fear extinction, again accompanied by increased global hippocampal levels of H3 and H4 acetylation [109]. Finally, a 14-day treatment with vorinostat was shown to exert the same effects following chronic corticosterone-induced stress as fluoxetine (see above, [96]), *i.e*. to prevent the upregulation of corticosterone-stress-induced biochemical changes including corticosterone-induced increased hippocampal HDAC2 gene and protein expression [96].

Systemic treatment with the specific HDAC class I inhibitor MS-275, targeting HDACs 1-3 and 8, effectively reduced the general decrease of acetylated histone H3 associated with inborn hyperanxiety in a high anxiety behavior (HAB) mouse model [110]. In the 129S1/SvlmJ (S1) mouse model characterized by deficient fear extinction, MS-275 furthermore promoted the dietary zinc restriction-induced rescue of deficient fear extinction and increased H4ac levels in the medial PFC and the amygdala [111]. Furthermore, long-term object-location memory - observed to be enhanced by administration of the broad-spectrum HDAC inhibitor trichostatin A after fear conditioning (see above) - could also be enhanced by administration of MS-275 [101]. Remote memory, not successfully extinguishable by reconsolidation paradigms alone, was successfully extinguished during extinction training by additional systemic, intraperitoneal administration of the class I HDAC inhibitor CI-994 one hour after memory recall [112].

The specific class I HDAC inhibitor RGFP963 was shown to enhance the consolidation of cued fear extinction when administered intraperitoneally five minutes after the last conditioned stimulus trial of extinction training and went along with decreased global H3ac and H4ac in the hippocampal dentate gyrus [113]. In the same study, the HDAC3-selective inhibitor RGFP966 increased H3ac in the basolateral amygdala and decreased global acetylation of histones H3 and H4 in the dentate gyrus when injected intraperitoneally one hour before extinction training, but – in contrast to the class I HDAC inhibitor RGFP963 (see above) - did not enhance consolidation of cued fear extinction [113].

Finally, blocking the histone lysine demethylase (HDM) 1 (LSD1) in the lateral amygdala by trans-2-phenylcyclo-propylamine (t-PCP) resulted in enhanced fear memory along with an increase in H3K9me2 levels [54].

#### 3.3.3 Experimental Compounds Targeting Writers (HMTs, HATs)

Inhibition of the H3K9me2 histone lysine methyltransferase G9a by UNC0224 in the lateral amygdala was linked to significantly less freezing behavior 24 hours after auditory fear conditioning and to decreased H3K9me2 levels [54]. Likewise, inhibition of G9a and GLP (G9a-like protein) by UNC0642 and A-366 in adult mice during paradigms to assess anxiety-like behavior such as the elevated zero maze was linked to a reduction in anxiety-like behavior and a general decrease of H3K9me2 methylation in the brain [98]. Pharmacological inhibition of the H3K9me3-specific histone methyl transferase SUV39H1 by means of ETP69, however, resulted in decreased levels of H3K9me3 in the hippocampus, but, however, to increased freezing behavior in a fear conditioning task [114].

Inhibition of p300/CBP by means of C646 or inhibition of p300/CBP and PCAF with Garcinol in the lateral amygdala shortly (one hour) after conditioning or fear memory retrieval significantly reduced freezing behavior 21 hours after fear conditioning *via* prevention of H3 acetylation in the lateral amygdala [115, 116]. Infusion of the combined p300/CBP histone acetyltransferase inhibitors C646 or Lys-CoA-Tat directly into the PFC post-extinction training led to significantly enhanced fear extinction [117]. Quite conversely, but rather consistent with the effects of HDAC inhibition as detailed above (3.3.2.), systemic administration of the PCAF inhibitor H3-CoA-20-Tat increased fear-like behavior in a contextual fear conditioning paradigm, and systemic administration of the PCAF activator *SPV106* resulted in increased fear extinction and decreased fear-like behavior [118].

### 3.4 Genetic Modifications Targeting the Histone Acetylation/Methylation Machinery

Another way of understanding the role of chromatin modifications in the pathogenesis of anxiety-, fear- and stress-related disorders and of discovering putative new epigenetic drugs in this field is by overexpressing or knocking down/knocking out specific genes of interest coding for the histone-related epigenetic machinery in rodent models.

Effects of specific HDAC classes were tested in the context of synaptic plasticity and memory formation with the help of conditional brain-specific *Hdac4* (Hdac4bko) and constitutive *Hdac5* knockout mice. In motor coordination, total locomotion and elevated plus maze tests, Hdac4b knockout mice displayed deficits in motor coordination as well as hyperactive and less anxiety-like behavior, which could not be observed in *Hdac5* knockout mice. Additionally, *Hdac4*, but not *Hdac5* knockout led to deficits in context-dependent learning and memory and displayed decreased LTP during theta burst stimulation on Schaffer collateral pathway, indicating a decrease in synaptic plasticity [119]. A study testing functional redundance of class I HDACs (HDAC1 and HDAC2) observed *Hdac2* but not *Hdac1* knockout mice – while not exhibiting a change in locomotor and anxiety-related behavior – to display enhanced performance in an attentional set-shifting task along with accelerated conditioned fear extinction [120], which is in line with enhanced fear extinction elicited by pharmacological HDAC inhibition as detailed above (3.3.2.). However, quite in contrast to the above-mentioned pharmacological studies on HDAC inhibition (3.3.2.), inhibition of HDAC1 *via* siRNA-mediated knockdown of *Hdac1* in the hippocampus was observed to lead to impaired fear extinction, while viral HDAC1 overexpression resulted in increased extinction of contextual fear memories along with a decrease of H3K9ac and an increase of H3K9me3 at the *c-Fos* promoter region in the hippocampus [121].

Localized knockdown of MeCP2, which acts as a transcriptional repressor in complex with histone deacetylases, in the basolateral amygdala led to a genome-wide increase in H3ac accompanied by increased anxiety-like behavior and deficits in cue-dependent fear conditioning [107].

Transgenic mice expressing an inhibitory, truncated form of the histone acetyltransferase (HAT) P300, lacking the HAT and activation domains, displayed impaired long-term recognition memory and contextual fear memory as well as a genome-wide decrease of H3ac in the forebrain [122]. Results of a subsequent study of postnatal knockdown of P300 in mice, with restriction to subregions of the forebrain, support the pivotal role of P300 in the hippocampus and cortical areas for the formation of long-term recognition memory and long-term contextual memory [123]. Further, knockout of the histone acetyltransferase *CBP* in the same regions – critical for the acetylation of lysines on histones H3, H4 and H2B – led to impairments in LTP and long-term memory of contextual fear and object recognition [124], which in summary is consistent with the findings of pharmacological p300/CBP inhibition by means of C646 or Garcinol to impair consolidation and reconsolidation of fear memory (see 3.3.3.).

In the nucleus accumbens shell, overexpression of G9a, a histone methyltransferase specific for H3K9me2, mainly associated with repressive transcription resulted in an increase in H3K9me2 levels and subsequently increased addiction- and anxiety-like behaviors [125]. *Vice versa*, it was found that knocking down G9a expression in the nucleus accumbens shell led to a decrease in addiction- and anxiety-like behaviors [126], which is in line with pharmacological HMT inhibition by UNC0224 [54] or UNC0642 and A-366 [98] also leading to a reduction in anxiety-like behavior (see 3.3.3.). Selective ablation of lysine (K) methyltransferase 2a (Kmt2a), a HMT specific for H3K4, which is linked to active transcription, but not the ortholog Kmt2b, in adult ventral striatum/nucleus accumbens neurons, however, markedly increased anxiety-like behavior in multiple behavioral paradigms [127].

In another knockdown study in mice, where KAP1, an essential cofactor of the epigenetic repressor family of zinc finger proteins, was deleted in the forebrain, mice exhibited heightened levels of anxiety-like behavior and stress-induced alterations in memory and spatial learning [128]. These behavioral changes were accompanied by dysregulation of hippocampal genes caused by a decrease of H3K9me3 and increased general acetylation levels of H3 and H4 [128].

Finally, a conditional-inducible H2A.Z knock-out resulted in enhanced fear memory in males, reduced stress-induced sensitization of fear learning in females and suppressed non-stressful memory in both sexes [129].

### 3.5 Histone Modifications in Human Fear-, Anxiety- and Stress-Related Disorders

In a sample of 17 war veterans suffering from PTSD as compared to 16 healthy controls, the genome-wide signal distribution profile of H3K4me3, H3K27me3, H3K36me3 and H3K9me3 in peripheral blood mononuclear cells differed significantly between patients and controls pointing towards a shift in the genomic location of these marks [130]. In another study by the same group, ChIPseq on six peripheral blood mononuclear cell samples of patients with PTSD compared with six samples of healthy controls, however, failed to reveal genome-wide alterations in H3K4me3 in PTSD, whereas on a gene-specific level H3K4me3 was found to be increased in PTSD patients around the promoter regions of the *WNT10B*, *WNT10A*, *WNT7A*, *DVL1* and *TCF7* genes [131]. Authors speculate that the discrepant findings on a genome-wide level might be due to the fact that in the latter study only about 40,000 cells were used for ChIPseq analyses [131], while the previous analyses were based on >10 million cells [130].

## 4 DISCUSSION

This review article provides an overview of the relevant available literature on histone modifications in animal models of fear, anxiety and acute/chronic stress, on pharmacological compounds and genetic modifications targeting the histone acetylation/methylation machinery in animals as well as on histone modifications in human fear-, anxiety- and stress-related disorders. Animal studies point towards several histone modifications to be dynamically altered in models of fear conditioning, retrieval and extinction, acute and chronic stress. Furthermore, histone modification writers/erasers are suggested to be involved in these processes. Only very few and underpowered studies have been published on histone modifications in humans, restricted to the phenotype of PTSD and to the investigation of peripheral biomaterial given the non-accessibility of human brain tissue *in vivo*.

As illustrated in Fig. (**2**), the most consistent findings imply genome-wide increased H3K4me3 in fear conditioning and fear memory retrieval as well as chronic stress in animal models and gene-specifically in human PTSD. Furthermore, several findings point to globally increased H3K9me2 in animal models of fear conditioning and fear memory retrieval, increased H3K9me3 following acute stress, and increased H3K27me2 in animal models of chronic stress. Decreased H3K27me3, however, was observed quite consistently in animal models of fear conditioning and acute stress, and decreased H3K9me1 following acute stress. Finally, whereas fear conditioning in most studies resulted in increased H3K9ac, H3K14ac and H4K5ac, animal models of chronic stress or offspring of chronically stressed animals showed decreased H3K9ac and H4K5ac. Most of these studies have reported genome-wide data without further specifying the genic/intergenic regions particularly enriched for shifts in histone modification patterns. Very few studies, however, have additionally provided a more in-depth investigation for instance by ChIP-Seq based unbiased peak calling followed by gene ontology analysis as well as by using publicly available microarray data or conventional mRNA analyses *e.g*., [47, 82]. These data – though presently still scarce – are essential to uncover yet unknown key genes and assess the functional, *i.e*. transcriptional relevance of altered histone modification signatures at particular genes in anxiety, fear or stress-related phenotypes. This will also aid in elucidating whether epigenetic changes constitute a mechanistic component in its own right or rather catalyze downstream processes in conferring anxiety-, fear- or stress-related symptoms, which, however, is not necessarily mutually exclusive (cf. [92]).

On a candidate gene level, BDNF is a promising candidate to be epigenetically studied in relation to fear, anxiety and stress as it plays a pivotal role in brain development, neural plasticity, memory formation and particularly the acquisition, consolidation and extinction of conditioned fear [49-51]. Accordingly, histone modifications have been found to be significantly altered in this context with for instance increased *Bdnf* promoter I H3K4me3 after fear conditioning, but decreased after extinction. Furthermore, increased *Bdnf* promoter IV H3 and H4 acetylation facilitating transcriptional activity was observed after fear conditioning but decreased H3ac was discerned at *Bdnf* promoters I, IV and VI after acute stress.

Furthermore, pharmacological inhibitors of CBP and PCAF, which act as histone acetyl transferases, have been reported to reduce freezing behavior after fear conditioning and enhance fear extinction [116-118]. These findings support previous evidence for a pivotal role of the transcription factor CREB (cAMP/Ca(2+) response element binding protein) in the formation of fear memories in general [132-134].

Despite these first promising findings on a genome-wide and candidate gene as well as pharmacological level it has to be noted that most studies are restricted to particular brain regions or even specific cell types and that the functional consequence remains to be elucidated. Also, some results could not be replicated or have even been contradicted by other studies, which, however, might be due to different paradigms applied across studies. A very important point to consider is the time course of histone modification dynamics in the context of fear, anxiety and stress. Several studies have shown that some effects observable one or two hours after a given event do not appear to persist after 24 hours, while other alterations seem to be detectable even after three weeks, which, however, might be specific to individual histone modifications and/or characteristic for particular paradigms. Finally, rodent models of fear, anxiety and stress most probably do not fully represent the complexity of human fear-, anxiety- and stress-related phenotypes [38] and thus might not accurately reflect their underlying epigenetic mechanisms.

The present review focused on the role of histone modifications and their regulators in fear-, anxiety- and stress-related phenotypes. However, other epigenetic mechanisms such as DNA methylation and non-coding RNAs have also been suggested to be involved in those states (*e.g*., [8, 9, 11, 14-23]. DNA methylation and histone modifications are highly interdependent with a significant bi-directional epigenetic crosstalk between histone-modifying and DNA methylation enzymes [135]. For instance, in a positive-feedback loop Methyl-CpG-Binding Domain Protein 1 (MBD1) bound to DNA methylation can recruit histone methylases like the SET Domain Bifurcated Histone Lysine Methyltransferase 1 (SETDB1) and thus provoke the methylation of H3K9. H3K9me3 in turn binds to DNMTs which leads to further DNA methylation and promotes heterochromatin formation [136]. It is hypothesized that DNA methylation might ’lock’ the repressive state previously gained by histone modifications [137]. Similarly, epigenetically relevant ncRNAs comprising micro-RNA (miRNA), small interfering RNA (siRNA), piwi-interacting RNA (piRNA) and long non-coding RNA (lncRNA) [138] mostly repressing gene transcription have been shown to influence heterochromatin formation in impacting histone modifications and DNA methylation [139, 140]. Thus, further research is warranted to understand the complex interplay of the whole array of epigenetic mechanisms in governing fear-, anxiety- and stress-related phenotypes. Furthermore, future studies should consider investigating mechanisms such as chromatin remodeling (*e.g*., changes in nucleosome positioning). First studies in that respect have suggested a role for aberrant regulation of CHD-type chromatin remodeling factors in learning and memory as well as contextual fear extinction in particular [141, 142], or of the ISWI-related remodeling enzyme SNF2H (also known as SMARCA5) in high anxiety behavior (HAB) mice [143].

A better understanding of the role of histone modifications and epigenetic mechanisms in general in conferring fear-, anxiety- and stress-related disorder risk might result in the definition of epigenetic biomarkers suited as both indicators and targets of preventive interventions [144]. As a prerequisite for the suitability of epigenetic marks as biosensors in humans, those markers would have to be stable enough to constitute a molecular signature of disease risk and to be measurable systemically as brain tissue is not accessible *in vivo* [145]. While many studies point towards a transient nature of chromatin modifications following learning or stressful experiences, there is some evidence for certain histone marks to persist following transcriptional events *e.g*., [146], and several studies suggest that certain peripheral DNA methylation marks might reflect central nervous system patterns (for review see 8; *in silico* databases such as BECon, IMAGE-CpG or Blood Brain DNA Methylation Comparison Tool), [147-149]. Given the general scarcity of studies on histone modifications in humans, to the best of our knowledge, no study so far has investigated the comparability of histone modification patterns in human peripheral material such as blood or saliva and human brain tissue. In this context, imaging tools such as positron emission tomography (PET) using tracers selective for histone deacetylases (*e.g*., [^11^C]Martinostat) or Lysine-Specific Demethylase 1 might prove to be highly useful in characterizing the histone modification machinery in the living human brain [150-155]. Apart from their potential as biomarkers of disorder risk, altered histone modifications might trigger downstream mechanisms such as mRNA, protein or structural changes eventually driving the pathogenesis of fear-, anxiety- or stress-related states. These downstream mechanisms remain to be investigated in future functional studies.

In analogy to the first studies demonstrating an intergenerational transmission of DNA methylation signatures associated with early life stress [156], the role of histone modifications in transporting the effects of stress in a transgenerational manner remains to be investigated. While many aspects of epigenetic intergenerational transmission in short-lived organisms, such as *C. elegans* and *D. melanogaster*, have been thoroughly described [29, 157, 158], the functions and mechanisms of epigenetic intergenerational transmission in mammals still remain poorly understood [29]. Recent studies in mammals suggest that histone post-translational modifications [159-161] could have important roles in the intergenerational transmission of information, but more work needs to be done for claiming causality [29, 162].

Pharmacological compounds targeting the histone acetylation/methylation machinery such as HDAC or HMT (G9a) inhibitors, which have been shown to possibly enhance fear-extinction processes or decrease anxiety-like behavior, respectively, in animal models, carry great promise also regarding the exposure-based and thus extinction-related treatment of human fear-, anxiety- and stress-related disorders. Such “epigenetic drugs” might constitute novel therapeutics in the treatment of anxiety and stress-related disorders by enhancing learning processes, fear extinction and neuronal plasticity [90-92, 163]. However, issues such as the lack of selectivity and specificity of currently available compounds, the partly antagonistic effects regarding fear memory consolidation and fear extinction in animal models and the risk of severe adverse effects when administered systemically remain to be solved before the field can proceed toward clinical trials in humans. As there is a burgeoning body of evidence for disease-associated DNA methylation patterns to “normalize” along with a response to psychotherapy [8], future studies might want to investigate whether histone modifications related to fear, anxiety or stress might also be malleable by psychotherapeutic interventions such as cognitive-behavioral therapy. On a related note, compounds targeting histone acetylation/methylation might be beneficial in augmenting extinction or extinction memory consolidation in a psychotherapeutic setting.

## CONCLUSION

The translation of epigenetic findings into human research is hoped to expand the present state of knowledge not only on the pathomechanisms underlying anxiety and stress-related disorders but also on their prognosis, diagnosis and treatment. However, considerable gaps of knowledge remain concerning the intricate role of epigenetic mechanisms in anxiety and stress-related disorders given the numerous obstacles in translating findings from animal models to human research [38], rendering clinical application a long way off. Still, as evident by the promising first advances in the field thus far as outlined above, further detangling the role of the epigenetic machinery in fear, anxiety and stress opens up novel avenues towards gaining a better understanding of mental disorder pathogenesis, their prevention as well as optimized treatment.

## Figures and Tables

**Fig. (2):**
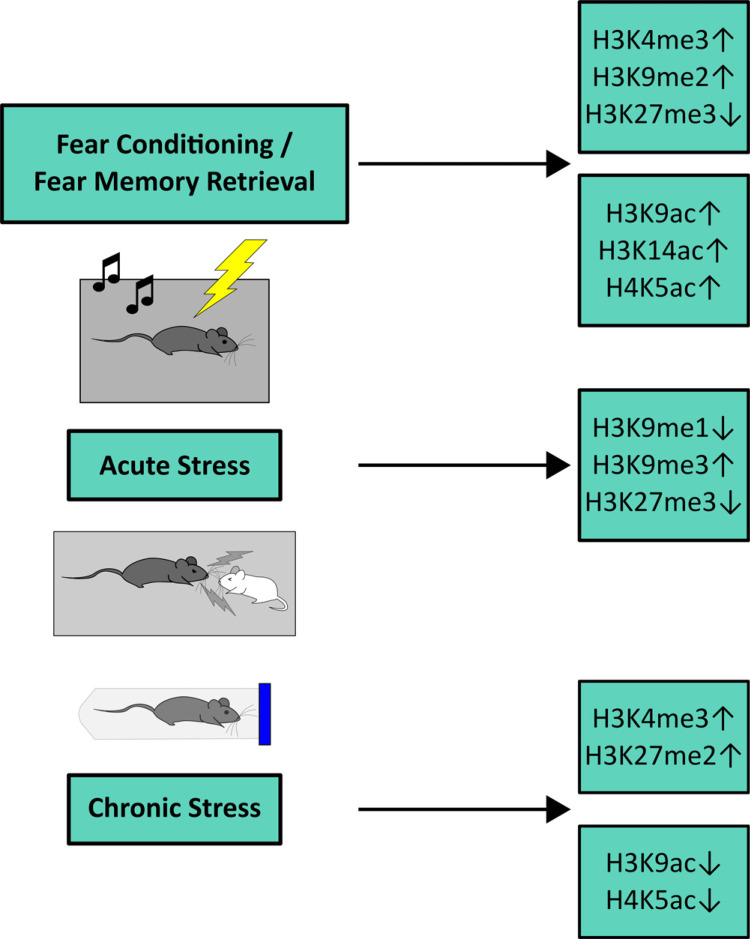
Graphical illustration of the currently most consistently identified genome-wide histone modifications in fear-/anxiety- and stress-related animal models. **Abbreviations**: ac: acetylation; H: histone (H3 or H4); K: lysine; Kx: amino acid position of lysine counting from N-terminus; me1: monomethylation, me2: dimethylation, me3: trimethylation.

**Table 1:** Overview of the most prominent histone modifications, their associated function and preferred location.

**Histone Modification**	**Function**	**Location**
H3K4me1	Activation	Enhancers
H3K4me3	Activation	Promoters, bivalent domains
H3K9me1	Activation	Promoters
H3K9me2, H3K9me3	Repression	Repetitive sequences
H3K27me2, H3K27me3	Repression	Promoters, developmental regulators, bivalent domains
H3K36me3, H3K79me2	Activation	Gene bodies
H3K9ac, H3K27ac	Activation	Enhancers, promoters
H3K14ac	Activation	Gene bodies
H4K5ac	Activation	TSS, Gene bodies
H4K16ac	Activation	Housekeeping genes

**Abbreviations:** ac: acetylation, H: histone (H3 or H4), K: lysine, Kx: amino acid position of lysine counting from N-terminus, me1: monomethylation, me2: dimethylation, me3: trimethylation, TSS: Transcription start site; also see 1.1.

**Table 2:** Dynamic histone modifications in animal models of fear/anxiety.

**Species**	**Paradigm**	**Measurement Post-training**	**Brain ** **Region**	**Genomic ** **Region**	**Histone ** **Modification**	**Additional Findings**	**References**
Rat	CFC and exposure alone	1 h	Hip (CA1)	Genome-wide	H3K9me2↑ H3K4me3↑	-	Gupta-Agarwal *et al.,* 2012 [45]
Rat	CFC and exposure alone	1 h	EC	Genome-wide	H3K9me2↑ H3K4me3↑	*-*	Gupta-Agarwal *et al.,* 2012 [45]
Rat	CFC and exposure alone	24 h	EC	Genome-wide	H3K4me3↓	-	Gupta-Agarwal *et al.,* 2012 [45]
Rat	CFC and exposure alone	1 h 24 h	Hip (CA1)	Genome-wide	H3K9me2↑ H3K9me2↓	-	Gupta *et al.,* 2010 [43]
Rat	CFC	1 h	Hip (CA1)	Genome-wide	H3K4me3↑	Returned to baseline after 24h	Gupta *et al.,* 2010 [43]
Mouse	CFC	1 h	Hip (CA1), ACC	Genome-wide	H3K9ac↑ H3K4me3↑ H3K27me3↓	DNAme↓ (ACC)	Halder *et al.,* 2016 [42]
Rat	CFC	1 h	Hip (CA1)	Genome-wide	H3K14ac↑	Returned to baseline after 24 h	Levenson *et al.,* 2004 [46]
Mouse	CFC	1 h	Hip	Genome-wide	H4K5ac↑	Lasting for 2-4 h depending on the amount of training	Park *et al.,* 2013 [47]
Rat	CFC	Immediately	Hip	Genome-wide	H2BK5ac↑ H3K9K14ac↑ H4K12ac↑	-	Bousiges *et al.,* 2013 [48]
Rat	CFC (SPS)	2 h	Hip	*Bdnf* promoter I and IV	H3ac↑ H4ac↑	*Bdnf* mRNA*↑*	Takei *et al.,* 2011 [52]
Rat	CFC	2 h	Hip (CA1)	*Bdnf* promoter IV *Bdnf* promoter II	H3ac↑, H3phospho-ac↑ H4ac↓	*Bdnf* exon IV mRNA*↑* DNAme at *Bdnf* exons I and IV↓ DNAme at Bdnf exon VI↑	Lubin *et al.,* 2008 [53]
Rat	Memory retrieval after CFC	1 h	Hip (CA1), BLA	Genome-wide	H3K4me3↑ (CA1) H3K9me2↑ (BLA)	H3K4me3 returned to baseline after 24 h 5hmC↑ (CA1)	Webb *et al.,* 2017 [44]
Rat	Memory retrieval after CFC	1 h	Hip (CA1)	*Npas4*	H3K4me3↑	5hmC↑	Webb *et al.,* 2017 [44]
Rat	Memory retrieval after CFC	30 days	ACC	*C-fos*	H3K4me3↑	5hmC↑	Webb *et al.,* 2017 [44]
Rat	AFC	1 h	Lateral amygdala	Genome-wide	H3K9me2↑	G9a mRNA↓	Gupta-Agarwal *et al.,* 2014 [54]
Rat	AFC	24 h	Lateral amygdala	Genome-wide	H3K9me2↓	-	Gupta-Agarwal *et al.,* 2014 [54]
Mouse	AFC	26 h	PFC	*Bdnf* promoter I and IV	H3ac↑	-	Bredy *et al.,* 2007 [55]
Mouse	AFC + extinction	2 h	PFC	*Bdnf* promoter I	H3ac↓	*Bdnf* I and IV mRNA*↑*	Bredy *et al.,* 2007 [55]
Mouse	AFC + extinction	2 h	PFC	*Bdnf* promoter IV	H4ac↑	*Bdnf* I and IV mRNA*↑*	Bredy *et al.,* 2007 [55]
Rat	Fear memory (5x CS+US)	2 h	PFC, amygdala	Genome-wide	H3ac↑ and H4ac↑ in BA, LA, CeM, CeL, PL-PFC	C-fos + CBP expression↑	Siddiqui *et al.,* 2017 [56]
Rat	Extinction	Immediately	PFC, amygdala	Genome-wide	H3ac↑ in IL-PFC and PL-PFC H4ac↑ in IL-PFC	C-fos + CBP expression↑	Siddiqui *et al.,* 2017 [56]
Rat	Fear memory (5x CS+US)	2 h	STMA, STLV (neurons)	Genome-wide	H3ac↑, H4ac↑	CBP↑	Ranjan *et al.,* 2017 [57]
Rat	Extinction	Immediately	STLP (neurons)	Genome-wide	H3ac↑, H4ac↑	CBP↑	Ranjan *et al.,* 2017 [57]
Rat	Immediate fear extinction (10 min)	2 h	IL-PFC, PLC	Genome-wide	PLC: H3K9ac↑ IL-PFC: H3K9ac↑	Retention of fear extinction↓ PLC: c-Fos↑ IL-PFC: c-Fos↑ PLC: CBP↑ IL-PFC: CBP↑	Singh *et al.,* 2018 [58]
Rat	Delayed fear extinction (24 h)	2 h	IL-PFC, PLC	Genome-wide	PLC: H3K9ac↑ IL-PFC: H3K9ac↑↑	Retention of fear extinction↑ PLC: c-Fos↑ IL-PFC: c-Fos↑ PLC: CBP↑ IL-PFC: CBP↑↑	Singh *et al.,* 2018 [58]

**Abbreviations:** ACC: Anterior cingulate cortex, AFC: Auditory fear conditioning, *Bdnf*: Brain Derived Neurotrophic Factor, BLA: Basolateral amygdala, CA1: First region in the hippocampal circuit, CBP: CREB-binding protein, CFC: Contextual fear conditioning, C-fos: Proto-oncogene, CS: Conditioned stimulus, EC: Entorhinal cortex, G9a: Euchromatin histone-lysine N-methyltransferase 2 (EHMT2), Hip: Hippocampus, IL-PFC: Infralimbic prefrontal cortex, Npas4: Neuronal PAS Domain Protein 4, PFC: Prefrontal cortex, PLC: Prelimbic cortex, SPS: Single prolonged stress, STLP: Antero-lateral bed nucleus of the stria terminalis, STLV: Lateral-ventral bed nucleus of the stria terminalis, STMA: Antero-medial bed nucleus of the stria terminalis, UCS: Unconditioned stimulus.

**Table 3:** Dynamic histone modifications in animal models of acute stress.

**Species**	**Paradigm**	**Measurement Post-stress**	**Brain ** **Region**	**Genomic ** **Region**	**Histone ** **Modification**	**Further ** **Results**	**References**
Rat	Restraint stress (30 min)	Immediately	Hip (DG, CA1)	Genome-wide	H3K9me3↑	-	Hunter *et al.,* 2009 [60]
Rat	Restraint stress (30 min)	Immediately	Hip (DG, CA1)	Genome-wide	H3K27me3↓	-	Hunter *et al.,* 2009 [60]
Rat	Restraint stress (30 min)	Immediately	Hip (DG, CA1)	Genome-wide	H3K9me1↓	-	Hunter *et al.,* 2009 [60]
Mouse	Acute restraint in chronic restraint stress model (21 days)	2 h	Hip (DG)	*Grm2* promoter	P300↑ --> H3K27ac↑	mGlu2 receptors↑	Nasca *et al.,* 2015 [61]
Mouse	Acute restraint in chronic restraint stress model (21 days)	24 h	Hip (DG)	*Grm2* promoter	P300↓ --> H3K27ac↓	mGlu2 receptors↓	Nasca *et al.,* 2015 [61]
Rat	Restraint stress (2 h)	2 h	Hip	*Bdnf* promoter I, IV, VI	H3ac↓	Bdnf mRNA↓	Fuchikami *et al.,* 2009 [62]
Rat	Restraint stress (2 h)	24 h	Hip	*Bdnf* promoter I, IV and IV	No sign. changes in H3ac	-	Fuchikami *et al.,* 2009 [62]
Rat	Social defeat (4 x 15 min)	30 min	Hip	Genome-wide	H3ac↑	-	Hollis *et al.,* 2010 [63]
Rat	Forced swim (30 min)	1 day	Hip (DG)	Genome-wide	H3ph↑	-	Bilang-Bleuel *et al.,* 2005 [64]
Rat	Predator stress (15 min)	1 day	Hip (DG)	Genome-wide	H3ph↑	-	Bilang-Bleuel *et al.,* 2005 [64]
Rat	Ether exposure (3 min)/ cold stress (4 h)	1 day	Hip (DG)	Genome-wide	No change in H3ph	-	Bilang-Bleuel *et al.,* 2005 [64]
Rat	Acute glucocorticoid treatment	1 h	Hip	Genome-wide	H3K9me3↓	Especially at B2 short interspersed nuclear elements	Bartlett *et al.,* 2021 [65]

**Abbreviations: ***Bdnf*: Brain Derived Neurotrophic Factor, CA1: First region in the hippocampal circuit, DG: Dentate gyrus, *Grm2*: Glutamate metabotropic receptor 2, Hip: Hippocampus, mGlu2: Metabotropic glutamate receptor 2, P300: E1A binding protein p300.

**Table 4:** Dynamic histone modifications in animal models of (sub-)chronic stress.

**Species**	**Paradigm**	**Measurement Post-stress**	**Brain ** **Region**	**Genomic Region**	**Histone ** **Modification**	**Further ** **Results**	**References**
Rat	Subchronic restraint stress (7 days)	1 day	Hip (CA1)	Genome-wide	H3K4me3↓	-	Hunter *et al.,* 2009 [60]
Rat	Subchronic restraint stress (7 days)	1 day	Hip (DG, CA1)	Genome-wide	H3K27me3↓	-	Hunter *et al.,* 2009 [60]
Rat	Subchronic restraint stress (7 days)	1 day	Hip	Genome-wide	H3K9me3↑	-	Hunter *et al.,* 2009 [60]
Rat	Subchronic restraint stress (7 days)	1 h	PFC	*Nedd4-1*	H3K9me2↓	HDAC↑	Wei *et al.,* 2016 [66]
Rat	CRS (21 days)	1 day	Hip (DG)	Genome-wide	H3K4me3↑	-	Hunter *et al.,* 2009 [60]
Rat	CRS (21 days)	1 day	Hip (DG)	Genome-wide	H3K9me3↓	-	Hunter *et al.,* 2009 [60]
Mouse	CRS (29 days)	-	Hip	Genome-wide	H3K9ac↓ H4K5ac↓	HDAC2↑ in Hip	Wu *et al.,* 2021 [67]
Mouse	Social defeat (10 days)	Immediately	PVN	Genome-wide	-	HDAC2↓	Elliott *et al.,* 2010 [68]
Mouse	Social defeat (10 days)	1 day	NAc	Genome-wide	-	HDAC5↓	Renthal *et al.,* 2007 [69]
Mouse	Social defeat (10 days)	10 days	NAc	Genome-wide	H3K9me2↓	G9a↓, Glp↓, Suv39h1↓	Covington *et al.,* 2011 [70]
Mouse	Social defeat (10 days)	24 h and 10 days	NAc	Genome-wide	H3K14ac↑	HDAC2↓	Covington *et al.,* 2009 [71]
Mouse	Social defeat (10 days)	28 days	Hip	*Bdnf* promoter III / IV	H3K27me2↑	Bdnf III and IV mRNA↓	Tsankova *et al.,* 2006 [72]
Mouse	Social defeat (10 days)	28 days	NAc	Genome-wide	H3K27me2↑	-	Wilkinson *et al.,* 2009 [73]
Mouse	Social isolation (10 days)	28 days	NAc	Genome-wide	H3K27me2↑ H3K9me2↑	-	Wilkinson *et al.,* 2009 [73]
Mouse	Social defeat (21 days)	Immediately	Hip	Genome-wide	H3K9ac↓	HAT↑ HDAC↓ in cortex	Montagud *et al.,* 2016 [74]
Mouse	Social defeat (21 days)	3 weeks	Hip	Genome-wide	H4K12ac↑ H3K4me3↑	HAT↑ in Hip	Montagud *et al.,* 2016 [74]
Rat	CVS(14 days)	1 day	Hip (DG, CA3)	Genome-wide	H4K12ac↓ H3 phospho-acetylation (Lys9/Ser10)↓	-	Ferland *et al.,* 2011 [75]
Mouse	CVS (15 days) + acute stress (30 min)	Immediately	Hip (DG)	Genome-wide	Chronic stress: H4K12ac↓ Acute stress: H4K12ac↑ Chronic + acute stress: H4K12ac↑	Chronic + acute stress: HPA activity↑	Ferland *et al.,* 2014 [76]
Rat	CVMS stress (14 days)	1 day	Amygdala, BNST	-	-	HDAC5↓ in males (CeA) CBP↑ in females (BNST)	Sterrenburg *et al.,* 2011 [77]
Rat	CVS (14 days)	1 day	Hip (DG, CA3)	Genome-wide	H4K12ac↓	-	Ferland *et al.,* 2011 [75]
Rat	CUS (28 days)	1 day	Hip	Genome-wide	H3K9ac↓	HDAC5↑	Liu *et al.,* 2014 [78]
Rat	CUS (21 days)	1 day	Hypothalamus	*Crhr1*	H3K9me3↓	-	Wan *et al.,* 2014 [79]
Mouse	Prenatal CUS (4 weeks)	Immediately	Hip	Genome-wide	H3K14ac↓ in offspring (stronger in females)	Spatial memory↓ in offspring	Benoit *et al.,* 2015 [80]
Mouse	Gestational stress (restraint and 24-h light disturbance)	-	Hip	Genome-wide	H3K14ac↓ in offspring	DNMT1↑, HDAC1↑, HDAC2↑ in offspring	Zheng *et al.,* 2016 [81]
Mouse	Repeated cross-fostering	72 days	Medulla oblongata	Genome-wide	H3ac↑ H3K4me3↑ H3K27me3↓	-	Cittaro *et al.,* 2016 [82]
Rat	PEPS (15 days)	1 day, 2 weeks, 2 months	Hip (CA3)	Genome-wide	H4ac↑ (long lasting for 2 months)	MeCP2 activation	Baĭdo *et al.,* 2009 [83]

**Abbreviations: ***Bdnf*: Brain derived neurotrophic factor, BNST: Bed nucleus of the stria terminalis, CA1: First region in the hippocampal circuit, CA3: Third region in the hippocampal circuit, CBP: CREB-binding protein, CeA: Central nucleus of the amygdala, *Crhr1*: Corticotropin-releasing hormone receptor 1, CRS: Chronic restraint stress, CUS: Chronic unpredictable stress, CVMS: Chronic variable mild, CVS: Chronic variable stress, DG: Dentate gyrus, DNMT1: DNA Methyltransferase 1, G9a: Euchromatin histone-lysine N-methyltransferase 2 (EHMT2), Glp: Euchromatin histone-lysine N-methyltransferase 1 (EHMT1), HAT: Histone acetyltransferase, HDAC: Histone deacetylase, Hip: Hippocampus, HPA: Hypothalamic-pituitary-adrenal axis, MeCP2: Methyl-CpG Binding Protein 2, NAc: Nucleus accumbens, PEPS: Prolonged emotional and pain stress, PFC: Prefrontal cortex, PVN: Paraventricular nucleus, Suv39h1: Suppressor of Variegation 3-9 Homolog 1.

**Table 5:** Pharmacological compounds targeting the histone acetylation/methylation machinery in animal models of fear, anxiety and stress.

**Species**	**Paradigm**	**Treatment**	**Measurement ** **Post-stress**	**Tissue**	**Genomic ** **Region**	**Histone ** **Modification**	**Further ** **Results**	**References**
**Valproic Acid and SSRIs/SNRIs**								
Mouse	Extinction	VPA	2 h	PFC	*Bdnf* promoter I and IV	H4ac↑	Long-term memory for extinction↑	Bredy *et al.,* 2007 [55]
Rat	Memory consolidation (2-day-FST)	VPA	Day 1	VLO, Hip	Genome-wide	-	Memory consolidation↑	Zhao *et al.,* 2013 [93]
Rat	Memory consolidation (2-day-FST)	VPA	Day 2	VLO, Hip	Genome-wide	-	Memory consolidation↑ Bdnf level in the VLO↓ phospho-ERK↓	Zhao *et al.,* 2013 [93]
Mouse	Memory reconsolidation (7 days) and extinction	VPA	1 day	-	-	-	Consolidation / reconsolidation and extinction↑	Bredy *et al.,* 2008 [94]
Rat	Predator exposure/ psychosocial stress regimen (for 40 days)	VPA	Immediately	PFC, whole blood	Genome-wide	-	Superoxide↑ (whole blood) ROS↑ (whole blood) HDAC↑ (PFC) normalized to naive levels by VPA	Wilson *et al.,* 2014 [95]
Rat	CUS (28 days)	VPA	1 day	Hip	Genome-wide	Prevents H3K9ac↓ H4K12ac↓	Prevents: HDAC5↑, anxiety- and depression-like behaviors↑	Liu *et al.,* 2014 [78]
Rat	CRS (21 days)	Fluoxetine (SSRI)	1 day	Hip (DG)	Genome-wide	Rescues H3K9me3↓	-	Hunter *et al.,* 2009 [60]
Mouse	Chronic CORT stress (21 days)	Fluoxetine (SSRI)	Behavior: 7 days following stress Biochemical: 1 day after behavioral tests	Hip	Genome-wide	-	Anxiety- & depression-like behaviors↓ Prevents: serum CORT and serum ACTH↑, MDA↑ and Glutathion↓, TNF-α and IL-1ß levels↑, NF-κB and HDAC2 gene expression↑, NF-kB p65, COX-2, HDAC2 and p-JNK protein expression↑	Kv *et al.,* [96]
Rat	CUS (28 days)	Venlafaxine (SNRI)	Immediately	Hip	Genome-wide	Prevents: H3K9ac↓, H3K14ac↓ and H4K12ac↓	Anxiety- & depression-like behavior↓ Prevents: HDAC5↑	Qiao *et al.,* 2019 [97]
Mouse	Fear extinction CUS (28 days)	Venlafaxine (SNRI)	1 day	Whole brain	Genome-wide	H3K9me2↓	Anxiety-like behavior↓	Wang *et al.,* 2018 [98]
**Experimental Compounds Targeting Erasers (HDAC, HDM)**								
Rat	Memory consolidation (CFC)	TSA/NaBu	1 h	CA1 (Hip)	Genome-wide	H3ac↑ and H4ac↑	Induction of LTP at Schaffer-collateral synapses↑	Levenson *et al.,* 2004 [46]
Mouse (nitric oxide synthase KO)	Fear memory consolidation	NaBu	Behavior: 1 h, 24 h and 7 days Biochemical analyses: 1 h	Hip, amygdala	Genome-wide	H3ac & H4ac↑	Contextual fear conditioning↑ (24 h & 7 days)	Itzhak *et al.,* 2012 [99]
Rat	Memory consolidation	TSA	1.5, 3 or 6 h post training	BLA	-	-	Memory consolidation↑, hippocampal Bdnf level↑	Valiati *et al.,* 2017 [100]
Rat	Memory consolidation	TSA or MS-275	Immediately after FC	Hip	-	-	Long-term object-location memory↑	Hawk *et al.,* 2011 [101]
Mouse	Anxiety (NIH; EZM)	NaBu acutely (3x on 1 day) or chronically (twice daily for 21 days)	30 min	Hip	Genome-wide	Acute: H4ac↑ Chronic: H4ac↓	Acute: anxiety↑ (only in NIH, not in EZM) Chronic: no anxiety-like behavior	Gundersen *et al.,* 2009 [102]
Rat	Stress (CORT micropellets in CeA for 15 days)	TSA bilaterally infused in CeA (at day 8, for 7 days)	1 day	CeA	Genome-wide	H3K9ac↑	Anxiety-like behavior↓ mechanical somatic threshold↑ c-Fos, CRF and SIRT6 expression↓ GR expression↑	Tran *et al.,* 2015 [103]
Mouse	Fear extinction	NaBu	Behavior: 1 h, 24 h and 7 days Biochemical analyses: 1 h	Hip, amygdala	Genome-wide	H3ac↑ (amygdala) H4ac↑	Speed of cued fear extinction↑	Itzhak *et al.,* 2012 [99]
Mouse	Fear extinction	NaBu (systemically), TSA (intrahippocampally)	Immediately	Hip	-	-	Fear extinction↑	Lattal *et al.,* 2007 [104]
Mouse	Fear extinction	NaBu (intrahippocampally)	30 min	Infralimbic cortex	Genome-wide	H3K14ac↑	Extinction levels↑ c-Fos expression↑	Stafford *et al.,* 2012 [105]
Rat	Spatial memory/ extinction (SPS)	NaBu	Immediately		-	-	Learning and memory↑ extinction of new memory↑	Farani *et al.,* 2020 [106]
Mouse	RTT model (Mecp2 forebrain KO mice)	SAHA	Western Blot: Immediately Cue-dependent: 4 hrs after context	BLA	Genome-wide	H3ac↑ H4ac↑	Anxiety-like behavior↑ cue-dependent memory ↓	Adachi *et al.,* 2009 [107]
Rat	CFC Fear extinction	SAHA	1 day 2 h	-Hip	-Genome-wide	-H3ac↑ H4ac↑	Fear extinction↑	Fujita *et al.,* 2012 [108]
Rat	SPS + CFC + extinction (2 days)	SAHA	24 h after extinction (behavior) 2 h after injection (histones & proteins)	Hip	Genome-wide	H3ac↑ H4ac↑	Fear extinction↑ Nr2B↑ CaMKII↑	Matsumoto *et al.,* 2013 [109]
Mouse	Chronic CORT stress (21 days)	SAHA treatment (14 days)	behavior: 7 days following stress biochemical: 1 day after behavioral tests	Hip	Genome-wide	-	Anxiety- & depression-like behaviors↓ Prevents: serum CORT and serum ACTH↑, MDA↑ and Glutathion↓, TNF-α and IL-1ß levels↑, NF-κB and HDAC2 gene expression↑, NF-kB p65, COX-2, HDAC2 and p-JNK protein expression↑	Kv *et al.,* 2018 [96]
Mouse	HAB *vs*. LAB	MS-275	2 h	Cingulate cortex neurons	-	Rescues H3ac↓	-	Sah *et al.,* 2019 [110]
Mouse	ZnR-induced fear extinction	MS-275	2 h	IL-PFC, BA neurons	Genome-wide	H4ac↑	Fear extinction↑	Whittle *et al.,* 2016 [111]
Mouse	Fear extinction (1 to 30 days) + exposure	CI-994 1 h post recall	1 day	-	-	-	Fear extinction↑	Gräff *et al.,* 2014 [112]
Mouse	Fear extinction	RGFP963 (5 min after extinction training)	1 h	BLA, DG	Genome-wide	H3ac↓ and H4ac↓ (DG)	Consolidation of cued fear extinction↑	Bowers *et al.,* 2015 [113]
Mouse	Fear extinction	RGFP966 (5 min after extinction training)	1 h	DG	Genome-wide	H3ac↑ (BLA) H3ac↓ and H4ac↓	-	Bowers *et al.,* 2015 [113]
Rat	Fear conditioning	t-PCP	24 h	LA	-	H3K9me2↑	Fear memory↑	Gupta-Agarwal *et al.,* 2014 [54]
**Experimental Compounds Targeting Writers (HMTs, HATs)**								
Rat	Fear conditioning	UNC0224	24 h	LA	-	H3K9me2↓	Fear memory↓	Gupta-Agarwal *et al.,* 2014 [54]
Mouse	Fear extinction	UNC0642 A-366	1 day	Whole brain	Genome-wide	H3K9me2↓	Anxiety-like behavior↓	Wang *et al.,* 2018 [98]
Rat	Memory consolidation	ETP69	24 h	Hip	Genome-wide	H3K9me2↓	Anxiety-related behavior↓ objection location memory↑	Snigdha *et al.,* 2016 [114]
Mouse	Fear extinction	Lys-CoA-Tat	24 h	IL-PFC	-	-	Fear extinction↑	Marek *et al.,* 2011 [117]
Mouse	Fear extinction	C646	24 h	IL-PFC	-	-	Fear extinction↑	Marek *et al.,* 2011 [117]
Rat	Memory reconsolidation after auditory fear conditioning	Garcinol	90 min	LA	Genome-wide	H3ac↓	Consolidation and reconsolidation↓	Maddox *et al.,* 2013 [115]
Rat	Memory reconsolidation	C646	90 min	LA	Genome-wide	H3ac↓	Consolidation and reconsolidation↓	Maddox *et al.,* 2013 [115]
Mouse	CFC + extinction (24 h later)	-	2h	IL-PFC	Genome-wide	H3K9me2↓	*MeCP2↑* HDAC2↓ PCAF↑	Wei *et al.,* 2012 [118]
Mouse	Fear extinction	SPV106 (PCAF activator) H3-CoA-20-Tat (PCAF inhibitor)	2h	IL-PFC	-	-	SPV106: anxiety-behavior↓ fear extinction↑ H3-CoA-20-Tat: anxiety-behavior↑	Wei *et al.,* 2012 [118]

**Abbreviations:** A-366: G9a/GLP Inhibitor, BA: Basal amygdala, *Bdnf*: Brain derived neurotrophic factor, BLA: Basolateral amygdala, C646: E1A binding protein p300 inhibitor, CA1: First region in the hippocampal circuit, CaMKII: Ca^2+^/calmodulin-dependent protein kinase II, CeA: central nucleus of the amygdala, CFC: contextual fear conditioning, *C-fos*: Proto-oncogene, CI-994: HDAC class I inhibitor tacedinaline, CORT: Corticosterone, CRF: Corticotropin Releasing Factor, CRS: Chronic restraint stress, CUS: Chronic unpredictable stress, DG: Dentate gyrus, ERK: extracellular-signal regulated kinases, EZM: Elevated zero maze, FC: Fear conditioning, FST: Forced swimming test, H3-CoA-20-Tat: PCAF inhibitor, HAB: High anxiety behavior, HAT: Histone acetyltransferase, HDAC: Histone deacetylase, HDM: Histone demethylase, Hip: Hippocampus, HMT: Histone methyltransferase, IL-PFC: Infralimbic prefrontal cortex, LA: Lateral amygdala, LAB: Low anxiety behavior, LTP: Long-term potentiation, Lys-CoA-Tat: E1A binding protein p300 inhibitor, MeCP2: Methyl-CpG Binding Protein 2, MS-275: HDAC class I inhibitor Entinostat, NaBu: Sodium butyrate, NIH: Novelty-induced hypophagia, Nr2B: N-methyl D-aspartate receptor subtype 2B, PCAF: P300/CBP-associated factor, PFC: Prefrontal cortex, RGFP963: HDAC class I inhibitor, RGFP966: HDAC3 inhibitor, ROS: Reactive oxygen species, RTT: Rett syndrome, SAHA: Suberoylanilide hydroxamic acid = vorinostat, SIRT6: Sirtuin 6, SNRIs: Serotonin and norepinephrine re-uptake inhibitors, SPS: Single prolonged stress, SPV106: PCAF activator, SSRIs: Selective serotonin re-uptake inhibitors, t-PCP: trans-2-phenylcyclopropylamine *=* histone lysine demethylase (HDM) 1 (LSD1) inhibitor, TSA: HDAC class I&2 inhibitor trichostatin A, UNC0224: selective G9a inhibitor, UNC0642: G9a/GLP Inhibitor, VLO: Ventrolateral orbitofrontal cortex, ZnR: Zinc restricted.

## LIST OF ABBREVIATIONS

ACC: Anterior Cingulate Cortex
BDNF: Brain-derived Neurotrophic Factor
DNA: Deoxyribonucleic Acid
HAB: High Anxiety Behavior
ncRNAs: Non-coding RNAs
PFC: Prefrontal Cortex
PTSD: Post-traumatic Stress Disorder
SAHA: Suberoylanilide Hydroxamic Acid
TFs: Transcription Factors
VPA: Valproic Acid
